# Human *SVIP* is sufficient to stimulate tubular lysosomes and extend healthspan in well-fed *Caenorhabditis elegans*

**DOI:** 10.17912/micropub.biology.001379

**Published:** 2024-11-01

**Authors:** Joshua P. Gill, Kathryn R. DeLeo, K. Adam Bohnert, Alyssa E. Johnson

**Affiliations:** 1 Biological Sciences, Louisiana State University, Baton Rouge, Louisiana, United States

## Abstract

Small VCP Interacting Protein (SVIP) is essential for maintaining a unique form of tubular lysosomes (TLs) in *Drosophila* . Although *Caenorhabditis elegans* do not have an annotated *SVIP* ortholog, expression of *Drosophila SVIP* in the *C. elegans* intestine induces TLs constitutively, increases autophagic activity, and extends healthspan. Here, we find that expression of the human ortholog of *SVIP* in the *C. elegans* gut causes similar physiological and phenotypic effects as *Drosophila SVIP* , albeit some effects were less pronounced. These results demonstrate that human *SVIP* can induce functional TLs in *C. elegans* but may be a weaker allele compared to *Drosophila SVIP* .

**Figure 1. Human SVIP induces TLs constitutively, increases autophagic activity, and improves healthspan when expressed in the C. elegans gut:**
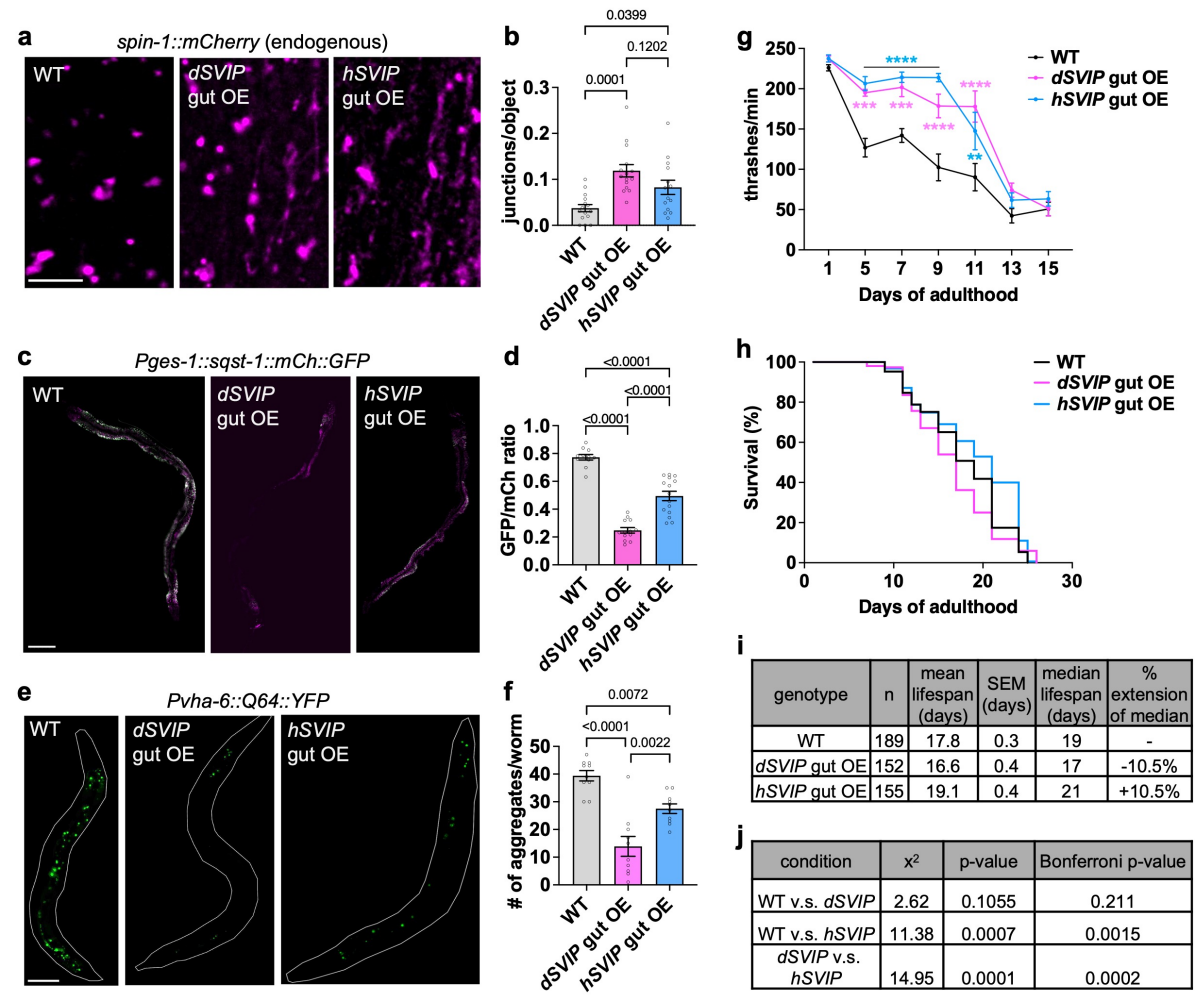
**a.** Endogenously tagged SPIN-1 ::mCherry in fed adult day 1 WT, *dSVIP* gut OE, and *hSVIP* gut OE worms (scale bar, 5μm). **b.** Quantification of lysosomal junctions/object in fed adult day 1 WT (n=15 worms), *dSVIP* gut OE (n=15 worms), and *hSVIP* gut OE (n=14 worms) worms (p-values indicated on graph). **c.** Gut-expressed SQST-1 ::mCherry::GFP in fed adult day 2 WT, *dSVIP* gut OE, and *hSVIP* gut OE worms (scale bar, 100μm). **d.** GFP/mCherry ratio in fed adult day 2 WT (n=11 worms), *dSVIP* gut OE (n=13 worms), and *hSVIP* gut OE (n=15 worms) worms (p-values indicated on graph). **e.** Gut-expressed Q64::YFP in fed adult day 1 WT, *dSVIP* gut OE, and *hSVIP* gut OE (scale bar, 100μm). **f.** Number of Q64::YFP aggregates in fed adult WT, *dSVIP* gut OE, and *hSVIP* gut OE worms on day 1 of adulthood (n=10 for each genotype; p-values indicated on graph). **g.** Thrashing rate of fed adult WT, *dSVIP* gut OE, and *hSVIP* gut OE worms (n=10 worms for each genotype; p-values on graph are comparisons to WT, **p<0.01, ***p<0.001, ****p<0.0001). **h.** Lifespan of fed adult WT, *dSVIP* gut OE, and *hSVIP* gut OE worms. **i.** Descriptive statistics for the lifespan comparison in Fig. 1h. **j.** Log-rank test results for lifespan comparisons in Fig. 1h.

## Description

Small VCP Interacting Protein (SVIP) was first described as an endogenous inhibitor of Endoplasmic Reticulum-Associated Degradation (Ballar et al., 2007) . Further work showed that SVIP recruits Valosin-Containing Protein (VCP) to lysosomes and is an essential protein for maintaining tubular lysosomal dynamic stability and autophagosomal-lysosomal fusion in *Drosophila* (Johnson et al., 2021; Johnson et al., 2015; Wang et al., 2011) . Additionally, overexpression of *SVIP* in *Drosophila* muscles causes an increase in tubular lysosome (TL) density (Johnson et al., 2021) . Significantly, TL induction correlates with improved animal health, and TL dysfunction has been linked to *VCP* -dependent diseases, underscoring the potential biomedical relevance of *SVIP* genes (Villalobos et al., 2023; Wall et al., 2021) .

There is no known *SVIP* ortholog present in the *Caenorhabditis elegans* genome; however, our lab previously generated a transgenic *C. elegans* strain that expresses a codon-optimized *Drosophila* *SVIP* gene ( *dSVIP* ) in the gut (Villalobos et al., 2023) . Although TLs are generally only stimulated in the gut of young-adult *C. elegans* under conditions of food limitation (Bohnert & Johnson, 2022; Dolese et al., 2022; Ramos et al., 2022) , expression of *dSVIP* in the gut was sufficient to induce gut TLs constitutively and heighten autophagic activity in young-adult, well-fed *C. elegans* (Ricaurte-Perez et al., 2024; Villalobos et al., 2023). The human genome encodes an *SVIP* ortholog ( *hSVIP* ), which has not been previously characterized in any animal model. In this study, we created a transgenic *C. elegans* strain that overexpresses a full-length codon-optimized human *SVIP* transgene in the gut and assessed its effect on TL induction, autophagic activity, lifespan, and healthspan. The purpose of this study was to determine if human *SVIP* has similar phenotypic and physiological effects as *Drosophila SVIP* .

To determine if human *SVIP* overexpression stimulates TL formation constitutively, we overexpressed *hSVIP* in the gut of a strain expressing the lysosomal membrane protein SPIN-1 endogenously tagged with mCherry. Subsequently, we imaged SPIN-1 ::mCherry in fed day 1 adults, a condition in which TLs are not normally induced (Villalobos et al., 2023) . While TLs were not robustly observed in young well-fed wildtype animals, overexpression of human *SVIP* induced constitutive TLs to a similar degree as overexpression of *Drosophila SVIP* ( **Figure 1 a-b** ). Next, we determined if gut *hSVIP* overexpression heightened autophagic activity as we observed previously with the *Drosophila* ortholog (Villalobos et al., 2023) . To do this, we imaged the autophagy receptor, SQST-1 , tandemly tagged with mCherry and GFP ( SQST-1 ::mCherry::GFP) (Villalobos et al., 2023) . This autophagy reporter relies on the principle that GFP fluorescence is sensitive to the acidity in the lysosomal lumen, while mCherry fluorescence is unaffected by the low pH environment (Klionsky et al., 2021) . Hence, low GFP fluorescence is indicative of more autophagic turnover. We imaged SQST-1 ::mCherry::GFP in the intestine of fed day 2 adults overexpressing human or *Drosophila SVIP* and compared autophagic turnover by quantifying the GFP/mCherry ratio. Overexpression of human *SVIP* in the gut decreased GFP fluorescence relative to control animals but not to the same extent as gut *dSVIP* overexpression (Figure **1c-d** ). Thus, human *SVIP* overexpression increases autophagic activity but not as strongly as the *Drosophila* transgene. Similarly, it has been shown that expression of *Drosophila SVIP* suppresses protein aggregation, and we were curious if the human transgene would do the same. To do this, we expressed the aggregation-prone protein Q64 (Morley et al., 2002) tagged with yellow fluorescent protein (YFP) in the gut and imaged fed day 1 adults. Indeed, human *SVIP* reduced protein aggregates in day 1 adults relative to control animals but again not to the same degree as the *Drosophila SVIP* transgene (Figure **1e-f** ). Thus, human *SVIP* overexpression heightens autophagic activity but not as strongly as *Drosophila SVIP* .

Next, we explored the physiological effects of human *SVIP* overexpression in the gut. It has been shown previously that expression of *Drosophila SVIP* in *C. elegans* improves healthspan independently of lifespan thereby decreasing the healthspan/lifespan gap (Villalobos et al., 2023) . We found that expression of human *SVIP* exhibited a comparable improvement in late age mobility, a strong proxy for healthspan (Hahm et al., 2015) , and had only a small effect on lifespan (Figure **1g-i** ). Thus, expression of human *SVIP* promotes healthy aging and increases healthspan similar to expression of the *Drosophila SVIP* gene.

Collectively, we found that the human *SVIP* transgene causes similar phenotypic and physiological effects as the *Drosophila* transgene. Both transgenes induced TLs, heightened autophagic activity, and increased healthspan. Interestingly, while human *SVIP* did not increase autophagic activity to the same extent as *Drosophila SVIP* , there was minimal difference in physiological effects between these two strains. Potentially, this could indicate that the maximum autophagic activity triggered by the *Drosophila* gene is not required to achieve the maximum physiological effects. Future comparative studies on other *SVIP* orthologs could be informative to designing synthetic SVIP peptides that would maximize the therapeutic potential of SVIP-based strategies aimed at improving healthy aging or combating age-related diseases.

## Methods

**Animal maintenance**

*C. elegans* were raised at 20ºC on Nematode Growth Medium (NGM) agar (51.3 mM NaCl, 0.25% peptone, 1.7% agar, 1 mM CaCl _2_ , 1 mM MgSO _4_ , 25 mM KPO _4_ , 12.9 µM cholesterol, pH 6.0) that were seeded with *E. coli* OP50 bacteria. Worms were synchronized by bleaching. Briefly, gravid hermaphrodites were vortexed in 1-2 mL bleaching solution (0.5 M NaOH, 20% bleach) for 3-5 minutes to isolate eggs, and eggs were washed three times in M9 buffer (22 mM KH _2_ PO _4_ , 42 mM Na _2_ HPO _4_ , 85.5 mM NaCl, 1 mM MgSO _4_ ) before plating on NGM plates seeded with OP50 bacteria. In all aging experiments, including the lifespan assay, adult worms were picked onto fresh OP50-seeded NGM plates every day to separate adults from progeny.

**Transgenic strain generation**

All strains used in this study were generated using standard genetic crosses or microinjection. For genetic crosses, transgenes expressing fluorescent proteins were tracked by stereomicroscopy. For microinjection, constructs were injected individually or in combination into the gonad of adult hermaphrodites, each at a concentration of 25 ng/µl. Integration of transgenes was achieved using UV irradiation, followed by >5 generations of outcrossing. The *dSVIP* transgene was generated in a previous study (Villalobos et al., 2023) , and the *hSVIP* transgene was generated as follows:

P *ges-1::hSVIP::unc-54 UTR:*

The coding sequence for human *SVIP* was codon-optimized for *C. elegans* expression using the *C. elegans* Codon Adaptor (Redemann et al., 2011) . This sequence, flanked by a 5' attB1 and a 3' attB2 sequence, was then synthesized as a gBlock by Integrated DNA Technologies:

GGGGACAAGTTTGTACAAAAAAGCAGGCTCAAAAaaaaATGGGGTTGTGTTTTCCTTGTCCGGGTGAGTCTGCTCCTCCAACCCCAGATCTTGAGGAGAAGCGGCTAAGCTCGCTGAGGCCGCTGAGCGCCGTCAAAAGGAGGCCGCTTCCCGTGGAATCCTTGACGTCCAATCCGTTCAAGAGAAGCGCAAGAAGAAGGAGAAGATCGAGAAGCAAATCGCCACTTCTGGTCCACCACCAGAGGGAGGACTCCGTTGGACCGTCTCCTAAACCCAGCTTTCTTGTACAAAGTGGTCCCC.

This *hSVIP* sequence was then cloned into the pDONR221 Gateway entry vector using BP clonase (ThermoFisher), and the insert was verified by DNA sequencing. Ultimately, pDONR221 *hSVIP* was combined with lab-stock plasmids pDONR P4-P1r P *ges-1* and pDONR P2R-P3 *unc-54* 3' UTR into the pDEST R4-R3 Gateway destination vector using LR clonase (ThermoFisher).

**Microscopy methods**

For *C. elegans* whole animal imaging, 4% agarose (Fisher Bioreagents) pads were dried on a Kimwipe (Kimtech) and then placed on top of a Gold Seal ^TM^ glass microscope slide (ThermoFisher Scientific). A small volume of 2 mM levamisole (Acros Organics) was spotted on the agarose pad. Worms were transferred to the levamisole spot, and a glass cover slip (Fisher Scientific) was placed on top to complete the mounting. Fluorescence microscopy was performed using a Leica DMi8 THUNDER imager, equipped with 10X (NA 0.32), 40X (NA 1.30), and 100X (NA 1.40) objectives and GFP and Texas Red filter sets.

**Image analysis**

Images were processed using LAS X software (Leica) and FIJI/ImageJ (NIH). Lysosome networks were analyzed using “Skeleton” analysis plugins in FIJI. Briefly, images were converted to binary 8-bit images and then to skeleton images using the “Skeletonize” plugin. Skeleton images were then quantified using the “Analyze Skeleton” plugin. Number of objects, number of junctions, and object lengths were scored. An “object” is defined by the Analyze Skeleton plugin as a branch connecting two endpoints, an endpoint and junction, or two junctions. Junctions/object was used as a parameter to quantify network integrity. For analyzing fluorescence intensity, the gut tissue was outlined using the free-draw tool in FIJI/ImageJ, and average fluorescence intensity of the outlined area was measured. For SQST-1 ::mCherry::GFP fluorescence ratio experiments, 50% laser intensity, 300 ms exposure time, and 100% Fluorescence Intensity Manager settings were used. For Q64 protein aggregate quantification, fluorescent aggregates were counted manually for each individual worm.

**Thrashing and lifespan assays**

Synchronous populations of animals were obtained by bleaching gravid adults (see animal maintenance), and worms in the late L4 larval stage were transferred to NGM plates seeded with OP50 bacteria. Throughout both assays, adult worms were transferred to fresh plates every day (to separate adults from their progeny) until reproduction ceased. For thrashing assays, individual worms were transferred into a drop of M9 buffer on an NGM plate, and the number of body bends were counted in a 1-minute period. For lifespans, dead worms were scored every 1-3 days. Worms that exploded, bagged, or crawled off plates were censored during analysis. Lifespans were analyzed using OASIS 2 software (Han et al., 2016) , and statistical significance was assessed using a log-rank test.

**Statistical Analyses**

Data were statistically analyzed using GraphPad Prism. For all experiments, data distribution was assumed to be normal, but this was not formally tested. For three sample comparisons, a one-way analysis of variance (ANOVA) with Tukey's multiple comparisons was used to determine significance (α = 0.05). For grouped comparisons, a two-way ANOVA with Šídák's multiple comparisons was used to determine significance (α = 0.05). Statistical significance of lifespan data was determined using a log-rank test.

## Reagents

**Table:** 

**Strain name**	**Genotype**	**Source**
Bristol N2	Wild type	Lab stock
COP2331	*spin-1 ( knu1010 [spin-1::mCherry::loxP::HygR::loxP]) V *	Villalobos et al., 2023 (by *In Vivo* Biosystems)
KAB58	*louIs5(* P *ges-1::dSVIP::unc-54 UTR +* P *odr-1::rfp) III*	Villalobos et al., 2023
KAB62	*louIs5(* P *ges-1::dSVIP::unc-54 UTR +* P *odr-1::rfp) III* ; *spin-1 ( knu1010 [spin-1::mCherry::loxP::HygR::loxP]) V *	Villalobos et al., 2023
KAB111	*louIs7(* P *ges-1::sqst-1::mCherry::gfp::unc-54 UTR) IV*	Villalobos et al., 2023
KAB143	*louIs5(* P *ges-1::dSVIP::unc-54 UTR +* P *odr-1::rfp) III;* *louIs7(* P *ges-1::sqst-1::mCherry::gfp::unc-54 UTR) IV*	Villalobos et al., 2023
KAB162	*louIs11(* P *vha-6::Q64::YFP + rol-6 ( su1006 )) *	This study
KAB170	*louIs13(* P *ges-1::hSVIP::unc-54 UTR +* P *odr-1::rfp)*	This study
KAB269	*louIs7(* P *ges-1::sqst-1::mCherry::gfp::unc-54 UTR) IV; louIs13(* P *ges-1::hSVIP::unc-54 UTR + Podr-1::rfp)*	This study
KAB281	*spin-1 ( knu1010 [spin-1::mCherry::loxP::HygR::loxP]) V; louIs13(* P *ges-1::hSVIP::unc-54 UTR +* P *odr-1::rfp)*	This study
KAB282	*louIs11(* P *vha-6::Q64::YFP + rol-6 ( su1006 )); * *louIs13(* P *ges-1::hSVIP::unc-54 UTR +* P *odr-1::rfp)*	This study
KAB341	*louIs5(* P *ges-1::dSVIP::unc-54 UTR +* P *odr-1::rfp) III;* *louIs11(* P *vha-6::Q64::YFP + rol-6 ( su1006 )) *	This study
